# Is Exposure to BMAA a Risk Factor for Neurodegenerative Diseases? A Response to a Critical Review of the BMAA Hypothesis

**DOI:** 10.1007/s12640-020-00302-0

**Published:** 2021-02-06

**Authors:** Dunlop RA, Banack SA, Bishop SL, Metcalf JS, Murch SJ, Davis DA, Stommel EW, Karlsson O, Brittebo EB, Chatziefthimiou AD, Tan VX, Guillemin GG, Cox PA, Mash DC, Bradley WG

**Affiliations:** 1 Brain Chemistry Labs, Institute for Ethnomedicine, Jackson, WY USA; 2 Lewis Research Group, Faculty of Science, University of Calgary, Alberta, Canada; 3 Department of Chemistry, University of British Columbia, Kelowna, BC Canada; 4 Department of Neurology, Miller School of Medicine, University of Miami, Miami, FL USA; 5 Department of Neurology, Dartmouth-Hitchcock Medical Center, Lebanon, NH USA; 6 Department of Environmental Science, Stockholm University, Stockholm, Sweden; 7 Department of Pharmaceutical Biosciences, Uppsala University, Uppsala, Sweden; 8 Qatar Natural History Group, Doha, Qatar; 9 Department of Biological Sciences, Macquarie University Centre for Motor Neuron Disease Research, Macquarie University, Ryde, Australia; 10 Nova Southeastern University, Fort Lauderdale, FL USA

**Keywords:** BMAA, Neurodegeneration, ALS, ALS/PDC, Cyanobacteria, Neurodegenerative diseases

## Abstract

In a literature survey, Chernoff et al. (2017) dismissed the hypothesis that chronic exposure to β-*N*-methylamino-L-alanine (BMAA) may be a risk factor for progressive neurodegenerative disease. They question the growing scientific literature that suggests the following: (1) BMAA exposure causes ALS/PDC among the indigenous Chamorro people of Guam; (2) Guamanian ALS/PDC shares clinical and neuropathological features with Alzheimer’s disease, Parkinson’s disease, and ALS; (3) one possible mechanism for protein misfolds is misincorporation of BMAA into proteins as a substitute for L-serine; and (4) chronic exposure to BMAA through diet or environmental exposures to cyanobacterial blooms can cause neurodegenerative disease. We here identify multiple errors in their critique including the following: (1) their review selectively cites the published literature; (2) the authors reported favorably on HILIC methods of BMAA detection while the literature shows significant matrix effects and peak coelution in HILIC that may prevent detection and quantification of BMAA in cyanobacteria; (3) the authors build alternative arguments to the BMAA hypothesis, rather than explain the published literature which, to date, has been unable to refute the BMAA hypothesis; and (4) the authors erroneously attribute methods to incorrect studies, indicative of a failure to carefully consider all relevant publications. The lack of attention to BMAA research begins with the review’s title which incorrectly refers to BMAA as a “non-essential” amino acid. Research regarding chronic exposure to BMAA as a cause of human neurodegenerative diseases is emerging and requires additional resources, validation, and research. Here, we propose strategies for improvement in the execution and reporting of analytical methods and the need for additional and well-executed inter-lab comparisons for BMAA quantitation. We emphasize the need for optimization and validation of analytical methods to ensure that they are fit-for-purpose. Although there remain gaps in the literature, an increasingly large body of data from multiple independent labs using orthogonal methods provides increasing evidence that chronic exposure to BMAA may be a risk factor for neurological illness.

## Introduction

$\beta$-*N*-methylamino-L-alanine (BMAA) is being studied by researchers throughout the world as a possible risk factor for progressive neurodegenerative illnesses. Originally discovered in cycad seeds in Guam, it is now known that BMAA occurs globally and is produced by cyanobacteria, diatoms, and possibly other organisms. Evidence for BMAA-triggered neurodegeneration is strongest among the Chamorro people of Guam where chronic exposure to BMAA through consumption of BMAA-contaminated foodstuffs, including cycad seed flour and flying foxes, appears to cause Guamanian amyotrophic lateral sclerosis/parkinsonism dementia complex (ALS/PDC). Chronic dietary exposure of nonhuman primates to total lifetime BMAA doses similar to those ingested by Chamorro villagers results in neuropathology in their brains similar to Guamanian ALS/PDC.

A key question is whether chronic exposure to BMAA constitutes a risk factor for human neurodegenerative diseases. One solution to protecting human populations from BMAA and its potential health consequences is to avoid exposure. National monitoring protocols in the USA would fall under the oversight of the Environmental Protection Agency (EPA). In 2017, Chernoff et al. (Chernoff et al. 2017) published a critical review of the BMAA hypothesis. Of the twelve authors of this review, several are government employees, some of whom work for the EPA. They wrote, “It is difficult to exaggerate the potential importance of the hypothesis that BMAA is a causal factor in the incidence of three devastating neurodegenerative diseases that affect millions of people worldwide. As the hypothesis begins to enter the public domain through popular science-oriented magazines *Discover Magazine* (McAuliffe 2011), *The Asian Scientist* (Lim 2012), and the *Scientific American* (Eplett 2015), the potential dangers associated with eating foods or living in environments that may contain high levels of BMAA are increasingly noted” (Chernoff et al. 2017 p. 13–14).

Unfortunately, Chernoff and colleagues selectively highlighted some negative papers and misstated or misinterpreted the results of others. For example, they claim that, “It should be noted that the toxicity of glutamate and BMAA was compared in vitro, and data demonstrated that glutamate was approximately tenfold more toxic than equimolar concentrations of BMAA (Chiu et al. 2012; Staton and Bristow 1997)”. However, this is inconsistent with the findings of Chiu et al. (Chiu et al. 2012) and misrepresents the data and conclusions of Staton and Bristow (Staton and Bristow 1997) who stated, “Glutamate was able to kill a significant number of cerebellar granule cells (CGC) after a 30-min exposure when viability was assessed 24 h later. BMAA and BOAA, however, required 24 or 48 h of exposure in which to cause a similar amount of cell death. This delayed type of death was indicative of an apoptotic-like process being involved.”

Questions regarding Chernoff et al. (2017) begin with the title which erroneously asserts that BMAA is a non-essential amino acid, which it is not. Essential amino acids are those of the twenty canonical amino acids used as building blocks for human proteins which cannot be synthesized by the human body, comprising histidine, isoleucine, leucine, lysine, methionine, phenylalanine, threonine, tryptophan, and valine. Non-essential amino acids are the eleven remaining 20 canonical amino acids that can be synthesized within the human body, e.g., alanine, arginine, asparagine, aspartic acid, cysteine, glutamic acid, glutamine, glycine, proline, serine, and tyrosine and thus are generally considered not essential in the diet. BMAA is neither type of amino acid. Instead it is a non-protein amino acid, an amino acid which is not preferentially used to make human proteins. Over 900 non-protein amino acids have been reported.

In the following consideration of the Chernoff et al. (2017) review, experts in this diverse and emerging field of study present data that support a link between BMAA and neurodegenerative disease, suggesting that further scientific investigation is warranted.

## The Detection and Quantification of BMAA

Chernoff et al. (2017, p. 14) write that “There are numerous sample preparation and analytical detection methods for BMAA; however, there has not been a standardization of these methods...” Bishop and Murch (2020) assessed 148 papers that were published on BMAA and examined BMAA quantification as reported in scientific papers up to April 2019; at least 8 more papers have been published since. Bishop and Murch (2020) reported that from 2003 to April 2019, 70% of the 148 published studies reporting BMAA analysis used reversed phase liquid chromatography (RPLC), 19% used hydrophilic interaction liquid chromatography (HILIC), and 11% used another analytical method, such as capillary electrophoresis or a commercially available BMAA enzyme-linked immunosorbent assay (ELISA) kit (Bishop and Murch 2020). BMAA was detected in one or more samples in 84% of the published studies. When categorized by methodology, 92% of RPLC studies, 57% of HILIC studies, and 71% of other studies reported BMAA in one or more samples of diverse origin (Bishop and Murch 2020). We have now updated these data to include published papers through August 2020 (Table 1).

**Table 1:** Methods used for BMAA analysis and percent of studies by method type that reported a positive result for BMAA for one or more samples. Studies are reviewed from the literature between 2003 and August 2020

	Method type			
	HILIC	RPLC	Other	Total
Number of studies	31	114	17	162
BMAA detected	18	107	12	137
% Total studies	19.1	70.4	10.5	
% BMAA detected	55	92	71	85

The BMAA detection literature is supported by several non-chromatography methods (Clausi et al. 2016; Faassen et al. 2013; Kerrin et al. 2017; Beri et al. 2018). However, we note that the currently available commercial ELISA is considered a flawed test by the scientific community generating many false positives and is not a good research tool in its present form (Faassen et al. 2013; Bláhová et al. 2017).

Chernoff et al. (2017) criticized the earlier papers that used liquid chromatography (LC) and recommended that these methods should be verified with liquid chromatography-mass spectrometry (LC-MS). However, some of the early papers they cited did in fact confirm their results using LC–MS or liquid chromatography with tandem mass spectrometry (LC–MS/MS) (Murch et al. 2004a, b; Pablo et al. 2009) and a careful reading of early BMAA papers indicated orthogonal methods to verify BMAA peaks were common.

**Fig. 1:**
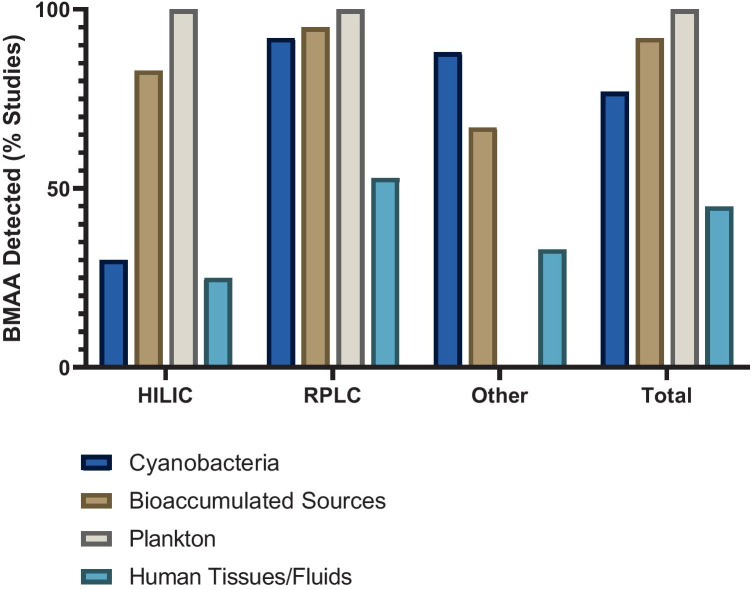
Percent of studies that reported a positive result for BMAA detection by sample type and method type. Cyanobacteria (dark blue bars) are from natural habitats, grown in vitro, or from dietary supplements. Bioaccumulated BMAA sources (brown bars) include human food sources and higher trophic level organisms. Plankton (grey bars) include phytoplankton (diatoms and dinoflagellates) or zooplankton. Human tissues and fluids (light blue bars) include brain (superior frontal gyrus, middle frontal gyrus, frontal cortex, temporal cortex or cerebellum), hair, blood, CSF and urine. Figure adapted from Bishop and Murch (2020) and studies reviewed from the literature between 2003 and August 2020.

Chernoff et al. (2017, p. 14) do state that later methods “focused on the use of mass spectrometry (LC/MS or LC/MS/MS) with better agreement regarding BMAA detection and to some extent quantitation.” We note that BMAA detection is matrix-dependent even within similar taxa. For example, Faassen et al. (2016) showed remarkably different recoveries between cyanobacterial species (only a 7% recovery for *Leptolyngbya* PCC 73110 but 78% for *Anabaena*) using the same protocol with HILIC methodology. In the 148 papers reviewed by Bishop and Murch (2020) there was good agreement in reporting of positive BMAA detection between different method types for most sample matrices. The largest discrepancy in detection arose from the study of cyanobacteria, where BMAA was detected in 95% of the RPLC studies of cyanobacterial matrices compared to only 25% of the HILIC studies (Fig. 1).

Chernoff et al. (2017) claimed that there has not been a “standardization” of BMAA methods, perhaps referring to method validation, which is the process to confirm that an analytical procedure employed is suitable for that intended use, in other words, fit for purpose (Glover et al. 2015; Banack 2020; Bishop and Murch 2020). There are many different guidelines that can be followed to validate a method and some of the organizations involved in method validation include the International Union of Pure and Applied Chemistry (IUPAC), US Pharmacopeia/National Formulary (USP/NF), the International Council for Harmonization (ICH), the US Food and Drug Administration (FDA), or the Association of Official Analytical Collaboration International (AOAC™). For example, the RPLC method using 6-aminoquinolyl-N-hydroxysuccinimidyl carbamate (AQC) derivatization has been validated for a cyanobacterial matrix by the AOAC™ guideline criteria (Glover et al. 2015), a fact which was not reported by Chernoff et al. (2017). The AOAC™ method using RPLC (Glover et al. 2015) was recently further validated using different instrumentation in a second laboratory analyzing not only a cyanobacterial matrix but also human cerebrospinal fluid, human blood plasma, and standardized mussel tissues from the National Research Council Canada (Banack 2020). This second laboratory validation of the AOAC™ method demonstrates the suitability of UPLC-MS/MS detection with AQC derivatization for the separation and quantification of BMAA and its isomers in a variety of matrices (Bishop et al. 2018). In addition to AQC, other derivatization methods such as EZ:Faast^™  ^and dansyl chloride are available to detect BMAA in complex matrices (Esterhuizen-Londt et al. 2011; Roy-Lachapelle et al. 2015, 2017; Main et al. 2018).

The majority of papers (86%) on BMAA methods since 2003 reported some performance characteristics for their method, including linearity, selectivity, precision, accuracy, etc. (Bishop and Murch 2020). However, only 43% of the papers reported the full set of performance characteristics necessary for a properly validated method for the matrix analyzed (Bishop and Murch 2020). In the future, research groups studying BMAA should ensure that their assay method for detection and quantification meets standard analytical method validation parameters. While it is unrealistic and unnecessary to suggest that everyone will use the same method and identical instrumentation, each method must be validated for a specific matrix in order to provide reproducible data. Any results from an analytical method that is validated properly for a specific matrix should be considered accurate, and the use of a single method for all types of analyses is not necessary.

Chernoff et al. (2017) also discussed common chromatography issues that could impede BMAA detection and quantification, such as separation of BMAA from its isomers and other interfering compounds. BMAA detection in complex matrices is difficult. While coelution is always a possibility, failures of mass spectrometry, such as ionization efficiency were not considered by Chernoff et al. (2017) (Glover et al. 2012; Banack and Murch 2018). BMAA forms dimers and adducts with common metals such as copper and zinc, making detection by mass spectrometry of BMAA in its native form (*m/z* 119 in positive ionization mode) difficult (Nunn et al. 1989; Glover et al. 2012). Derivatization of BMAA can prevent the spontaneous formation of metal adducts during analysis since the amine groups of the molecule are protected and it increases the mass to charge ratio (*m/z*) of the ion, which results in greater accuracy and selectivity (Banack and Murch 2018). Although the use of several characteristic ion transitions in MS/MS improves method accuracy, the choice of transition ion used may provide different results (Baker et al. 2018). Many groups have reported incomplete separation of BMAA using HILIC-MS/MS from its isomer β-amino-*N*-methyl-alanine (BAMA). Since analysis of the BMAA isomer BAMA began ca. 2012, earlier studies using mass spectrometry may have failed to obtain adequate selectivity of BMAA over its isomers (Jiang et al. 2012; Beach et al. 2015, 2018; Réveillon et al. 2015, 2016; Foss et al. 2018; Li et al. 2018)*.* We note that BMAA and BAMA are widely separated by retention times using AQC derivatization (Banack 2020). The *m/z* transition 119 > 102 in underivatized methods has shown inconsistent results in the literature (Baker et al. 2018; Beach et al. 2018; Li et al. 2018; Tymm et al.  2019). Authors have used additional ion separation steps, such as differential mobility spectrometry (DMS), which acts as an ion filter by separating ions in a matrix based on their mobility in high or low electrical field strength, to adequately separate BMAA from BAMA but this requires a skilled analyst (Beach et al. 2015, 2018).

Discrepancies in results may also arise from different linear analysis range and limits of detection (LOD) between methods. When detected in environmental samples, BMAA is often in low concentration at or slightly below the LOD, which could explain non-detection by less sensitive methods. Absence of evidence is not necessarily evidence of absence; failure to detect does not equal absence of analyte. Some earlier HILIC studies reported linear ranges from 10 to 1000 ng/mL while the linear ranges for several RPLC studies were as low as 0.8–100 ng/mL, which could have resulted in discrepancies in results (Rosén and Hellenäs 2008; Réveillon et al. 2014; Glover et al. 2015; Faassen et al. 2016; Lage et al. 2016)*.* HILIC methods generally report a higher LOD than RPLC methods, with one study reporting a LOD 150 times higher for HILIC compared to the RPLC methods (Baker et al. 2018; Bishop and Murch 2020; Yan et al. 2017). The increased molecular weight of derivatized BMAA facilitates identification of the precursor and product ions by MS/MS, leading to higher sensitivity, and RPLC columns are generally more robust than HILIC columns (Faassen et al. 2016; Yan et al. 2017). Certain HILIC columns tend to have a short lifetime which can result in a loss of signal of up to 100% over a 6-week period, resulting in extremely low recoveries and inaccurate and imprecise results (Tymm et al. 2019). It is noteworthy that an attempt to validate the analysis of BMAA using a ZIC®-HILIC column in a cyanobacterial matrix failed to meet AOAC™ guideline criteria in “selectivity, accuracy, precision, repeatability, and demonstrated poor sensitivity and lack of ruggedness during chromatographic runs” (Tymm et al. 2019). This study concluded that “analysis of a cyanobacterial matrix for BMAA using a ZIC®-HILIC column is not fit for purpose” and therefore “not suitable for detection or quantification of BMAA or its isomers in cyanobacteria or other related biological matrices” (Tymm et al. 2019). Furthermore, the quantification of BMAA using RPLC and AQC derivatization requires careful attention to total amino acid concentrations to ensure a complete derivatization reaction. Failure to do so can result in a dramatic under-estimation of BMAA concentration and is likely the reason for some concentration discrepancies found in the literature (Cohen 2012).

Although Chernoff et al. (2017) stated that there are inconsistencies between the forms of BMAA extracted and analyzed in published studies (i.e., free BMAA, protein bound BMAA, or total BMAA), most of the 148 papers reviewed by Bishop and Murch (2020) reported which fraction of BMAA was analyzed. After the 2004 discovery that BMAA can occur in a protein bound or associated form (Murch et al. 2004a, b), 89% of studies have reported the use of acid hydrolysis, either to find total BMAA in samples or have separately analyzed the free, soluble-bound, and protein-bound forms of BMAA (Bishop and Murch 2020). BMAA has been found in varying concentrations in these different fractions according to sample type, and as long as results are reported accurately, this should avoid confusion in the literature (Cheng and Banack 2009; Murch et al. 2004a, b; Rosén et al. 2016).

Matrix effects which differ using different methods, true biological variability using different samples from different ecosystems, and low analyte concentrations likely cause the largest discrepancies in results within similar matrices (Banack and Murch 2018; Bishop and Murch 2020). Ecologically speaking, variation in temporal and spatial scales is a very common and pervasive phenomenon and should not be used as a disqualifier. The study of ecology aims to understand the scales under which ecological phenomena and patterns occur, and how the local climate affects ecological interactions (i.e., biogeography, assembly rules; Schneider 2001; Sanmartín 2012; Andersson et al. 2014). Furthermore, differences in sample preparation methods as simple as drying affect total BMAA recovery within samples (Banack 2020). Chernoff et al. (2017) wrote that the use of standard addition or isotope dilution will compensate for these issues. While prohibitive costs and lack of availability of isotopically labeled BMAA standards limited their use in earlier studies, stable BMAA internal standards are available and more researchers currently employ this technique for quantitation, where appropriate. This, along with increased availability of standard reference materials will increase reproducibility of results between laboratories. Comparison of the same sample between laboratories has been conducted in the past (e.g., Li et al. 2010; Mondo et al. 2014; Réveillon et al. 2014; Davis et al. 2019; Banack 2020) and further inter-laboratory comparisons of this kind will provide additional confirmation of BMAA analysis and quantification across laboratories and should be encouraged.

The use of internal standards, however, does not always account for analyte loss during sample preparation. In a HILIC comparison study of three sample matrices (cyanobacteria, animal, brain), authors used a D_3_-BMAA internal standard, yet they were unable to explain why D_3_-BMAA spiking of the cyanobacterial strain *Leptolyngbya* resulted in very low recoveries of 7–21% while D_3_-BMAA spiking with the zooplankton *Daphnia magna* resulted in very high recoveries of 141% (Faassen et al. 2016). Poor recovery of less than 10% was found for the same sample matrices after solid-phase extraction (SPE) cleanup and derivatization with AQC [Section S.2.3 Supplemental Data (Faassen et al. 2016)]. Meanwhile, a validated RPLC method with AQC derivatization demonstrated adequate recovery (between 104.8 and 107.5%) of BMAA in a cyanobacterial matrix at three different analyte spike concentrations (0.122–0.183 ng/mL) (Glover et al. 2015). This indicates that differences in sample preparation, instrumentation, and operator expertise contribute to loss of target analyte and cannot be fully accounted for by use of an internal standard. Researchers must ensure that analyte losses during sample preparation are minimized and that the recovery is within an acceptable range with spiking levels close to the lower limit of quantitation (LOQ) before the method used can be considered valid for that matrix.

## ALS/PDC Is a Neurodegenerative Disease with Similarities to Sporadic ALS

Chernoff et al. (2017) assert that amyotrophic lateral sclerosis/parkinsonism dementia complex (ALS/PDC) is a disease unique to Guam and the Western Pacific and therefore not of interest to the rest of the world. They support this claim by highlighting a unique retinopathy that may not be present in other populations that do not get ALS/PDC, “The identity of the ALS/PDC found in Guam is a fundamental issue because if it is a separate disease, the role of BMAA in Guam and the Kii Peninsula, even if shown to be a causal factor in ALS/PDC, may be of limited concern worldwide” (Chernoff et al. 2017, p. 14).

We note that there are definite differences in the pathology between Guamanian ALS/PDC and sporadic ALS (sALS), but there are also many similarities (Hirano et al. 1966; Oyanagi et al. 1994; Galasko et al. 2002). Importantly, Guamanian ALS/PDC is associated with tar DNA-binding protein 43 (TDP-43) proteinopathies similar to frontotemporal lobar degeneration with ubiquitin positive inclusions (FTLD-U) with/without motor neuron disease (MND) as well as ALS, and neocortical or hippocampal TDP-43 pathology which distinguishes controls from disease subjects in these entities better than tau pathology. The spectrum of TDP-43 proteinopathies should be extended to include neurodegenerative cognitive and motor diseases affecting the Chamorro population of Guam (Geser et al. 2008).

We would not necessarily expect an isolated island ethnic group, with likely very different genetic makeup, to respond in the same manner to BMAA or any other neurotoxin. Therefore, it is not surprising that the pathology looks different. Phenotypically, Guamanian ALS looks very similar to ALS in the rest of the world. The whole idea of pleiotropy suggests a vast array of neurodegenerative processes resulting from a single genetic mutation (Bradley and Mash 2009; Bradley et al. 2019).

The literature is replete with examples where a genetic mutation within the same gene can cause different distinct and independently recognized diseases (see Bradley and Mash 2009 for examples). Mutations in the progranulin gene have been shown to lead to different neurological disease diagnosis (Alzheimer’s disease, Parkinson’s disease, FLTD-U, and progressive supranuclear palsy) in different individuals (Brouwers et al. 2007; Spina et al. 2007; Wider et al. 2008). Frontal temporal dementia, Parkinson’s disease, Alzheimer’s disease, and ALS are all likely related and environmental exposures can potentially lead to a variety of neurodegenerative processes. As we learn more about risk factors for ALS and the pattern of accumulation of those risk factors, we realize that BMAA is only one of the causative factors for ALS (Chiò et al. 2018; Bradley et al. 2019). If one considers that ALS may be related to a series of environmental triggers, one of them being BMAA, this pallet of toxins is likely different in Guam than in the rest of the world.

## Misrepresentation of the Flying Fox Hypothesis

The Chernoff et al. (2017) section on flying foxes is a clear example of their critique misrepresenting the literature.

“Banack, Murch, and Cox (2006) reported on a similar number of dried skin samples from an unspecified source using HPLC-FD for quantification, and BMAA levels were considerably lower than those reported in 2003 (479 µg/g vs 3556 µg/g). This difference necessitates additional investigation since the magnitude of flying fox BMAA levels played a major role in the contention that consumption of these animals was a critical factor in its role as a cause of ALS/PDC” (Chernoff et al. 2017, p. 18).

First, the source of the flying fox samples from Banack et al. (2006) was clearly stated in the first paragraph of the materials and methods section 2: “Tissue samples from 21 accessioned flying fox specimens on deposit at the Museum of Vertebrate Zoology, University of California, Berkeley (MVZ), and the United States National Museum, Smithsonian Institute, Washington, DC (USNM), were analyzed.” Furthermore, the 21 specimens presented in this analysis included not only dried skins but also fluid-preserved (formalin fixed and stored in 70% alcohol) and frozen specimens. The concentration of BMAA in individual specimens and various tissues from *Pteropus mariannus mariannus* (the Guam flying fox) ranged from 47 to 1202 µg/g (see Table 2 in Banack et al. 2006), and a hair sample was found to be 1859 µg/g (see Table 1 in Banack et al. 2006). One would expect the BMAA concentration in ~ 50-year-old tissues to vary between animals, tissue types, and preservation methods. Natural variation in cycad consumption patterns of individual animals is expected due to seasonal variation, spatial variability, and individual animal preference. The BMAA concentrations reported in the larger sample set of Banack et al. (2006) when compared with Banack and Cox (2003a) represent real-world natural variability and are accurate. LC–MS verification of BMAA peaks was conducted in this study (Banack et al. 2006). We note a recent publication on BMAA within flying fox tissues published since the Chernoff et al. (2017) review (in which Chernoff is a co-author) failed to find BMAA in these tissues (Foss et al. 2018). This attempt to analyze flying fox tissue for BMAA used a ZIC®-HILIC column which, as previously mentioned, has been shown to be unreliable and not fit for the purpose of BMAA analysis in biological matrices (Foss et al. 2018; Tymm et al. 2019). Foss et al. (2018) reported co-elution of BMAA and BAMA using that method and reported a wide range of spike recoveries for total BMAA analysis in flying fox tissues using hydrolysis and SPE (68 ± 21%), further indicating that the method used is not fit for the matrix analyzed.

With regard to the importance of the consumption of flying foxes as a causative factor in the etiology of Guamanian ALS/PDC, several factors should be considered. 

(1) The concentration of BMAA in flying foxes is indeed high relative to BMAA concentrations in most organisms from other worldwide ecosystems (e.g., Jonasson et al. 2010; Brand et al. 2010; Christensen et al. 2012; Mondo et al. 2012, 2014; Field et al. 2013; Masseret et al. 2013; Al-Sammak et al. 2014; Banack et al. 2014, 2015; Jiang et al. 2014; Réveillon et al. 2016; Hammerschlag et al. 2016; Scott et al. 2018; Lance et al. 2018).

(2) Eating flying foxes was considered by the Chamorro people to be an important part of their cultural heritage and in the more isolated parts of Guam, where the people lived a more traditional lifestyle, incidence rates of ALS/PDC were higher (Kisby et al. 1992; Lemke 1992; Sheeline 1993; Plato et al. 2002; Monson et al. 2003; Borenstein et al. 2007). This desire to uphold cultural traditions in Guam by eating flying foxes has led to the extinction of one flying fox species, *Pteropus tokudae*, and the near extirpation of the other species, *Pteropus mariannus mariannus* (Wiles and Payne, 1986; Bräutigam and Elmqvist 1990; Lujan 1992; Wiles 1992; Cox and Sacks 2002; Monson et al. 2003).

Chernoff et al. (2017) offer misleading information concerning the consumption of flying foxes by the Chamorro people, selectively reporting information suggesting limited consumption from sources such as Lemke (1992) while ignoring repeated data in the same Lemke paper concerning the massive impact of hunting for human consumption on the flying fox populations.

Consider, for example, the statements by Chernoff et al. (2017, p. 19) from the Lemke (1992) publication written to suggest that humans have limited flying fox consumption.

“Lemke (1992) observed that consumption of flying foxes occurred at social events and religious holidays, not as a dietary staple. Generally, one bat was ingested in a meal for two people, but given their scarcity, a single animal may be employed in a meal for several individuals”.

versus other statements from the same Lemke (1992, Pg. 135–139) document:

“Since the 1970s fruit bat populations in the northern Mariana Islands have seriously declined because of commercial hunting for human consumption ...Even in the face of fruit bat extinctions local residents demand an oppportunity to hunt bats and enjoy this cultural delicacy... It is important to recognize why fruit bats are immensely sought after … Thompson (1945) listed bats as an important food source in her monograph on ancient Chamorro culture … Fruit bats, because of their distinct taste, odor, and perhaps rarity, top the list of desirable food items to serve at special occasions. When fruit bats are prepared, none of the animal is wasted. Recipes implore that bats do not require skinning or eviscerating, simply washing the fur is sufficient (Rody 1982) ...The fur, meat, viscera, and wing membranes are eaten. … The negative effects of commercial hunting on CNMI bat populations have been expressed by numerous authors (Wheeler and Aguon 1978; Brunner and Pratt 1979; Ralph and Sakai 1979; Wheeler 1980; Payne 1986; Wiles and Payne, 1986;

and similar published data not cited by Chernoff:

“Extensive ethnobotanical interviews were conducted with Chamorros in Umatac, Merizo, Agat, and Rota concerning their dietary practices. Chamorros were asked to list their favorite foods… Chamorros in these villages overwhelmingly identified flying foxes (*fanihi* in the Chamorro language) as the most salient and desired traditional food. When flying foxes were abundant, informants indicated eating up to seven to nine flying foxes per meal. The results from our interviews are consistent with Sheeline’s (1991) survey of more than 200 Chamorros in which 53% of those interviewed like to eat flying fox. In addition, 55% of respondents indicated that other people within their households ate flying foxes, 49% indicated that eating flying fox was an important part of being a Chamorro, and 19% of those surveyed could distinguish different species of flying fox by taste. In our interviews, some Chamorros told us that nothing in the world “tastes as good as *fanihi* … a single evening hunt at a flying fox roost could result in harvests of dozens of flying foxes (Graham, 1992). As a result, flying fox consumption accelerated, particularly in the aftermath of World War II.” (Cox et al. 2007, Pg. 270–272).

While acknowledging the importance of over-hunting as a “major factor during the first half of the 20th Century”, Chernoff et al. (2017, p. 19) make the claim that “the extirpation of *P. mariannus* has been due, to a significant extent, to the accidental introduction of the brown tree snake (*Boiga irregularis*) at some point during the 1950s.” This ignores detailed evidence that beginning in 1975 over 220,000 dead flying foxes were imported to Guam in a 15-year period in order to fill the nearly insatiable demand for flying fox flesh once *Pteropus mariannus* populations in Guam had crashed due to hunting (Bräutigam and Elmqvist 1990). Importation of flying foxes to Guam caused their endangerment throughout the islands of the Pacific. The dangers that the loss of these keystone pollinators and seed-dispersers posed to island ecosystems resulted in proscriptions against international trade in flying foxes through the Convention in International Trade and Endangered Species (CITES) (Cox et al. 1991; Cox and Elmqvist 2000; Allen-Wardell G et al. 2008). Instead, Chernoff et al. (2017) report peripherally related information, such as the brown tree snake which distracts from the central points: (a) Chamorros relish eating flying foxes and as a result ate a lot of them with heavy commercial hunting beginning in the mid to late 1960s and peaking in the mid to late 1970s (Lemke 1992); (b) the rapid consumption of local flying fox populations predated the documented Guamanian ALS/PDC prevalence data which peaked in 1965 and is precisely predicted by the data reporting BMAA as a “slow toxin” with a delay in the time from exposure to disease manifestation (Garruto et al. 1980, 1981; Spencer 1987; Cox and Sacks 2002); and (c) a preference for traditional Chamorro food is the only factor which epidemiologists have been able to correlate with an increased risk for ALS/PDC in Guam (Reed et al. 1987; Cox and Sacks 2002; Cox et al. 2007; Borenstein et al. 2007; Banack et al. 2010).

(3) Other animals in the Guam ecosystem consume cycad seeds and contribute to chronic BMAA dietary exposure of the Chamorro people (Banack et al. 2006; Banack and Murch 2009; Banack et al. 2010). Furthermore, the Chamorro people directly eat flour made from the gametophyte of cycad seeds which is an additional source of BMAA in their diet (Kisby et al. 1992; Cheng and Banack 2009). The culinary process of washing toxins out of cycad flour is helpful in removing free BMAA but leaves behind protein-bound BMAA in considerable quantities (Cheng and Banack 2009). 

(4) The BMAA content of a single serving of flying fox soup, prepared by Chamorros, was empirically measured to be 79 mg of BMAA (100 g of muscle + 1 L broth, Banack et al. 2006). This fact coupled with specific information about the consistency of flying foxes in the Chamorro diet suggests that the consumption of BMAA in the Chamorro diet was both frequent and widespread (Banack and Cox 2018). 

(5) Chronic low concentrations of BMAA ingested orally creates neuropathology in non-human primates remarkably similar to ALS/PDC (Cox et al. 2016; Davis et al. 2020). Furthermore, the concentrations of BMAA found in non-human primates chronically dosed with BMAA and in Chamorro tissues were within similar ranges (Banack and Cox 2018). Combined, these five factors make it difficult to dismiss the causative role of BMAA in the etiology of Guamanian ALS/PDC. Therefore, the concluding discussion from Chernoff et al. (2017, p. 33) relative to this topic is not accurate:

“The original examples of high BMAA levels in flying foxes were based on a small number of preserved, dehydrated animal skin samples (Banack and Cox 2003a) that cannot be used to extrapolate to fresh, hydrated tissues. The magnitude of these BMAA levels has not been replicated, and this is also true of similar levels reported in fish (Brand et al. 2010).”

The literature clearly demonstrates that 23 flying foxes have been examined for BMAA concentrations in two separate experiments. These experiments are complete with supporting orthogonal methods to detect BMAA and the concentrations reported are comparable to fresh, hydrated tissues (Banack and Cox 2003a; Banack et al. 2006). An examination of the literature also shows numerous publications from multiple labs of BMAA concentrations in fish, shark, and invertebrates using a plethora of techniques that corroborate the potential for worldwide chronic human consumption of BMAA from aquatic sources (e.g., Jonasson et al. 2010; Jiang et al. 2014; Jiao et al. 2014; Lance et al. 2018) and potential human exposure through terrestrial food sources (Andersson et al. 2013, 2018).

Chernoff et al. (2017) also dismiss the literature on dietary exposures of indigenous people outside of Guam to BMAA. For example, they report that “Roney et al. (2009) noted BMAA in the cyanobacterial species *Nostoc flagelliforme* that is used to make a soup used for celebrations. It is difficult to draw firm conclusions from the data presented because of the 18 samples listed, BMAA was detected and measured in 4 (0.027–0.659 μg/g), not quantified in one, not detected in 6, and not tested in 7.” (Chernoff et al. 2017, p. 10).

The Roney et al. (2009) paper noted that this heterogeneity in the presence of BMAA led to the discovery that counterfeit forms of *Nostoc flagelliforme* have been introduced to the Chinese market. BMAA could not be detected in any of the four counterfeit samples tested but was detected in four of the six real samples tested. All of these samples were purchased from Chinese markets. As Roney et al. (2009, p. 45–48) wrote:

“We detected the neurotoxic, non-protein amino acid BMAA and its isomer 2,4 diamino butyric acid (DAB) in some, but not all, samples of *fa cai* noodles collected both in China and in samples purchased in the United States and the United Kingdom. The extraordinary heterogeneity of the presence or absence of BMAA in these samples (Table I) led us to microscopic examination of the samples: one set of soup samples clearly consisted of mucilaginous tubes surrounding long chains of cyanobacteria, but other samples had no observable cyanobacteria and were instead composed of granular material (Figure 2) that stained positive for starch with KI... Since the counterfeit *fa cai* is of such high quality that it can be detected only by fluorescence microscopy or LC/mass spectrometry, it is astonishing, given the high price of *fa cai*, that many samples we purchased consisted of real *Nostoc flagelliforme* cut with the counterfeit product (Table I) – why go to the effort to add the expensive real product to the artificial noodles? One possible answer could be that real *fa cai* soup has a detectable physiological impact on experienced consumers since it contains the excitotoxin BMAA, a feature that perhaps makes other neurotoxic meals desirable."

## Animal Studies and Environmental Exposures

Chernoff et al. (2017) dismiss a replicated study that showed that chronic dietary exposure to BMAA triggers in non-human primates the formation of neurofibrillary tangles (NFTs) and $\beta$-amyloid deposits similar to those found in the brain tissues of Chamorro villagers who died from Guamanian ALS/PDC. They stated that, “Lemere et al. (2004) examined the effects of a vaccine on β-amyloid deposits in vervet monkeys and detected no NFTs in animals that were 20+ years old, which is inconsistent with findings in Cox et al. (2016) that found NFTs in the control animals.” (Chernoff et al. 2017, p. 21).

Their statement suggests that Chernoff et al. (2017) are unclear about tau immunohistochemistry (IHC) visualization, with background AT8 clone antibody staining, which can occur even when no developed NFTs are present. In clinical neuropathology, NFTs are usually detected using the AT8 clone antibody, which recognizes the hyperphosphorylated form of tau at residues 202 of serine and 205 of threonine. This is an excellent antibody, but like all antibodies, it can yield non-disease-relevant immunopositivity in individuals. In Cox et al. (2016), the AT8 clone antibody and IHC were used to visualize NFTs across the vervet cerebral cortex and several deep gray matter nuclei in 40-micron-thick brain sections. The vervet brain sections used in this study were 8 times thicker than diagnostic neuropathology sections. Although there was background immune staining in control animals, they did not observe NFTs in control animals. In BMAA-dosed animals, there were various stages of NFT formation, which mirrors the human condition for early disease. For example, in Alzheimer’s disease, NFTs emerge in the brain as pre-tangles which stain positive with AT8 antibody (early, not associated with dementia), fibrillary tangles (mature), and ghost/tombstone tangles associated with dead neurons. NFTs are also associated with plaques, commonly called neuritic plaques, which are highly correlated with dementia. An important observation in this report was the clear evidence of NFT development in BMAA-dosed animals (Fig. 2e–p in Cox et al. 2016) which had not previously been observed in vervets.

Cox et al. (2016) reported global digital counts for AT8+ immunostaining per brain region which included: mature NFTs as well as neuropil threads, pre-tangles, ghost tangles and background IHC cell staining. The latter type was abundantly observed in the placebo group and were never classified as mature NFTs in the paper. This was clearly stated in the first sentence of the legend of Fig. 3 in Cox et al. (2016, p. 6): “Median counts for density of AT8 IHC positive staining inclusions plus NFT per brain area by treatment type.”

A recent follow-up study by Davis et al. (2020) shows the mean value of AT8+ IHC NFT density across seven cortical brain regions of the same vervet primates from Cox et al. 2016 (Fig. 8b in Davis et al. 2020). These data clearly show that chronic dietary exposure to BMAA increases AT8+ IHC NFT density threefold in the vervet brain. The co-administration of L-serine reduced BMAA toxicity by 40% (Fig. 8b in Davis et al. 2020). This result further highlights that any brain regions of vervets dosed with BMAA with AT8+ immunostaining below < 50 counts are considered at the level of the control and/or predominantly background staining.

Chernoff et al. (2017, p. 22) suggests that, “A definitive association of BMAA exposure levels and disease is necessary before conclusions may be reached concerning BMAA and human neurodegenerative diseases.” The data from well-controlled and replicated experiments where non-human primates were chronically administered BMAA orally with food (Cox et al. 2016; Banack and Cox 2018; Davis et al. 2020) demonstrates that the neuropathology found in the Guam disease of ALS/PDC was re-created in the experimental model.

## Dosing Paradigms

Chernoff et al. (2017, p. 19) dismiss a multitude of peer-reviewed, published, scientific data with their statement, “Studies using routes of exposure that are not relevant to environmental exposures may be reliable strategies to answer mechanistic or relative tissue deposition questions, but extremely difficult or impossible to reach firm conclusions on applicability of such data to the oral route that is the primary means of BMAA environmental exposure.” We note that oral administration of BMAA to mice over a month of age did not produce behavioral or neuropathological changes (see review by Karamyan and Speth 2008), and this may be due to species specific or age-related differences (Scott and Downing 2019). However, a chronic oral administration of BMAA, in relevant concentrations to human exposure, given to a non-human primate model did reproduce the precise neuropathology of ALS/PDC and directly addresses this critique (Cox et al. 2016; Banack and Cox 2018; Davis et al. 2020).

Chernoff et al. (2017) argue that previous studies do not facilitate determination of environmental BMAA concentrations that are dangerous to human health. The oral route is likely the primary way of BMAA exposure although recent studies have revealed that BMAA also can be transferred to the brain via the olfactory pathways (Pierozan et al. 2020). However, as the oral bioavailability of BMAA is high in both rats and primates (Duncan et al. 1991, 1992), subcutaneous administration is considered relevant for investigating effects of the toxin in an animal model. Subcutaneous exposure of neonatal rats on post-natal day (PND) 9– 10 to the lowest BMAA doses tested impaired adult learning and memory function without any distinct acute or long-term histopathological changes in the brain (Karlsson et al. 2011), whereas 460 mg/kg induced progressive neurodegeneration in the hippocampus, including increased levels of α-synuclein, TDP-43, fibril formation, and neuronal calcification (Karlsson et al. 2009b, 2011, 2012, [Bibr CR117][Bibr CR117]). Neonatal exposure to all three BMAA doses (40, 150, and 460 mg/kg) altered the level of neuropeptides important for brain development (Karlsson et al. 2013). Recent in vitro studies further demonstrate that early-life exposure to BMAA can impair neuronal stem cell proliferation and differentiation (Pierozan and Karlsson 2019). Corroborating research from a second lab demonstrates that a single low dose of BMAA administrated subcutaneously early in life (50 mg/kg on PND 3) causes behavioral alterations, $\beta$-amyloid deposition, and neuronal loss in the hippocampus of adult rats (Scott and Downing 2019). These data suggest that more attention should be given to the potential adverse effects of BMAA exposure on brain development. Follow-up studies should include even lower doses and oral exposure to identify the no observed adverse effect level (NOAEL) and further elucidate underlying mechanisms. Furthermore, Chernoff et al. (2017) fail to consider co-exposure issues, e.g., individuals exposed to cyanobacterial blooms likely face more than one cyanotoxin, or in the case of simultaneous red tides and cyanobacterial blooms, multiple cyanotoxins plus brevetoxins (Metcalf et al. 2020) or even methyl mercury, whose toxicity BMAA potentiates in vitro (Rush et al. 2012; Mondo et al. 2014; Hammerschlag et al. 2016)*.* Also, the magnitude of toxicity may be different among BMAA isomers at different ratios, e.g., BMAA is more toxic at lower concentrations than *N*-(2-aminoethyl)glycine (AEG) to brine shrimp (Metcalf et al. 2015) while in vitro AEG is much more neurotoxic (Schneider et al. 2020)**.** In desert water and soils, BMAA has been found and is highly and positively correlated with both AEG and DAB concentrations (Cox et al. 2009; Craighead et al. 2009; Chatziefthimiou et al. 2016).

## BMAA in AD and ALS Brains

BMAA has been positively identified in some but not all brain areas of humans who suffered from ALS/PDC, Alzheimer’s disease, ALS, and Parkinson’s disease (Murch et al. 2004a, b; Bradley and Mash 2009; Pablo et al. 2009). Samples were fully blinded to the analytical labs and pair matched controls were included with all studies. The positive detection rate was 38%, and the majority of samples analyzed per batch were asymptomatic controls for which no BMAA was detected. These studies used a method with a complete in-house Single Lab Validation including performance measures of selectivity, linearity and range, accuracy, precision, sensitivity (detection limits and quantification limits), repeatability, and stability. A subset of the method performance parameters was included in the publications. Each of the publications had confirmatory data using orthogonal detection methods. BMAA was found only in very small amounts in both asymptomatic control tissues and in individuals who died with Huntington’s disease (Pablo et al. 2009).

There have been other published papers that used different methods and different tissues which were unable to detect BMAA in human brain tissues for many reasons largely related to the brain regions sampled, sample preparation techniques, detection methods employed, and sensitivity (Perry et al. 1990; Montine et al. 2005; Snyder et al. 2009, 2010; Combes et al. 2014). It is important to remember that the inability to detect an analyte does not mean that it is not present.

Chernoff et al. (2017) erroneously stated that the authors failed to report quantitative evidence of BMAA in ALS spinal cords. In fact, BMAA was detected in spinal cord tissues of individuals with ALS (Pablo et al. 2009). This is key to linking toxicity with lower motor neuron disease. More recent experimental data (Davis et al. 2020) provide direct evidence of BMAA damage to the spinal cord of non-human primates with chronic dietary exposure to BMAA which appears to mimic disease pathology associated with early-stage ALS; this result is similar to that obtained from intrathecal administration of BMAA in a murine model (Yin et al. 2014).

As stated in Chernoff et al. (2017) access to rare tissues makes replication of many of the experiments discussed difficult. The authors make the case that there is a lack of consistency between results for analysis of brain tissues of patients suffering from ALS and AD. There are obvious limitations to obtaining these rare samples and only nine studies are cited. Three of these studies reported a positive result for BMAA in brain tissues of ALS or AD patients (Murch et al. 2004a, b; Pablo et al. 2009), while five of the studies did not detect BMAA in any of the brain tissues analyzed (Perry et al. 1990; Montine et al. 2005; Snyder et al. 2009, 2010; Combes et al. 2014) and one study detected BMAA in the CSF of one patient with ALS and two asymptomatic patients (Berntzon et al. 2015). Upon inspection of the table summarizing these data (Table 3 in Chernoff et al. 2017), the largest difference between these studies is analytical method used. However, there are some errors within the data presented by Chernoff et al. (2017) in their table, e.g., the authors stated that the quantification method used by Snyder et al. (2009) was high-performance liquid chromatography with mass spectrometry (HPLC-MS) when it was gas chromatography-mass spectrometry (GC-MS) with ethyl chloroformate derivatization. The authors also claim that Murch et al. (2004a, b) analyzed only the protein-bound BMAA fraction, when in fact these papers also analyzed and reported the free fraction (Cox et al. 2003; Murch et al. 2004a, b).

As described in detail earlier, analytical methodology for BMAA detection is complicated and all methods must be fit-for-purpose for the sample matrix and concentration range analyzed (Bishop and Murch 2020; Banack and Murch 2018). The earliest study analyzing BMAA in rare tissues used an automatic amino acid analyzer for detection of BMAA (Perry et al. 1990); however, no performance characteristics specific to BMAA detection were reported. This is problematic since BMAA is found in much lower concentrations than canonical amino acids and can co-elute with amino acids such as ornithine on these types of instruments. This, along with other methods such as GC–MS, have not been used frequently in the last 10 years making comparison to current literature difficult. GC–MS may present some difficulties for BMAA analysis, as native BMAA is not volatile and must be derivatized before analysis (Guo et al. 2007; Snyder et al. 2009, 2010). Snyder et al. (2010, 2009) used methodology for derivatization of BMAA via ethyl chloroformate or a trimethylsilation reagent developed from Guo et al. (2007) before GC–MS or comprehensive two-dimensional gas chromatography time-of-flight mass spectrometry (GCxGC-TOFMS). However, this requires electron ionization (EI) which is a hard ionization technique that breaks molecules into many fragments (Harris and Lucy 2020, p. 586). Not enough subsequent studies were done to adequately determine whether all fragments of derivatized BMAA were detectable using this technique.

Chernoff et al. (2017) argues that if BMAA is indeed a globally occurring compound, it should be present to some extent in all tissue samples as with other organic pollutants such as polychlorinated biphenyls (lipid soluble), perfluorooctanoic acid (water soluble), and perfluorooctanesulfonic acid (water soluble). These chemicals they mentioned have a much larger molecular weight than BMAA and would not be metabolized in the same way in tissues. Further, unlike BMAA, they do not have a biologically relevant structure and are unlikely to be mistaken for canonical amino acids or act as a glutamate receptor agonist (Rao et al. 2006; Dunlop et al. 2013; Glover et al. 2014; Dunlop and Guillemin 2019; Han et al. 2020).

The mechanisms of neurodegenerative diseases are only partially understood and while growing evidence demonstrates that exposure to BMAA may increase the risk of disease, understanding of the degree of exposure that constitutes risk is currently nascent and requires further investigation. Gene-environment interactions may influence the development of the clinical-pathological phenotype expressed as a specific form of neurodegenerative disease in some individuals, but not others (Bradley et al. 2019). While a direct link has been demonstrated between the ingestion of BMAA and the onset of Guamanian ALS/PDC neuropathology in non-human primates (Cox et al. 2016; Banack and Cox 2018; Davis et al. 2020), the same causative link has not yet been proven between BMAA and progressive neurodegenerative disease outside of Guam. While our understanding of the genetic basis of ALS risk is increasing rapidly, with over 50 genes now recognized as being associated with the disease (Mejzini et al. 2019), the relationship between genetic risk, environmental exposure, and phenotype remains relatively unstudied (Al-Chalabi and Hardiman 2013). Mathematical modelling of sporadic ALS suggests six “hits” or steps are required to trigger the onset of disease (Al-Chalabi et al. 2014; Vucic et al. 2019), and this is reduced to three when a gene mutation is included (Chiò et al. 2018). Importantly, absence of evidence is not necessarily evidence of absence, since BMAA may also be present in an undetected form, such as an adduct complexed to metal ions or other biological molecules, or in a dimerized form which would change its mass effectively thwarting detection by mass spectrometry (Nunn et al. 1989; Glover et al. 2012).

## ALS Clusters Are Linked with Cyanobacterial Blooms

Correlations between proximity to cyanobacterial blooms and incidence of ALS have been established (Fiore et al. 2020; Torbick et al. 2018, 2014) with one study also reporting that BMAA was present. A significant cluster of ALS was identified in the Hérault district in Southern France, surrounding the Thau lagoon and this correlated with both RPLC (MS/MS) and HILIC identification of BMAA, AEG, and DAB in mussels and oysters with similar quantification results (Masseret et al. 2013; Réveillon et al. 2014). The authors proposed ingestion as the most likely route of exposure since the local population are heavy consumers of shellfish all year around (Réveillon et al. 2015). Several additional studies link ALS or ALS/PDC clusters or disease within a patient with the presence of dietary BMAA (Banack and Cox 2018; Banack et al. 2006, 2014, 2015; Banack and Murch 2009; Field et al. 2013).

## BMAA Is Produced by Cyanobacteria

Chernoff et al. (2017) considered the possibility of BMAA production in cyanobacteria and discussed three main points, being (1) the association of BMAA with cyanobacteria, (2) the degree of BMAA production by cyanobacteria, and (3) production of BMAA and DAB by organisms other than cyanobacteria.

Concerning point 1, this was largely resolved by Spácil et al. (2010) who assessed the presence of BMAA in an axenic strain of *Leptolyngbya* from the Pasteur Institute, Paris, France (PCC 73110). The analysis of such an axenic PCC strain gives confidence that in the absence of eukaryotes and other organisms, the LC–MS detection of 109.4 µg/l would indicate that this cyanobacterial strain can produce BMAA (Spácil et al. 2010). Furthermore, the approach adopted by Metcalf et al. (2017) combining underivatized chiral separation with derivatization of the collected fractions of *Nostoc* isolates from cycad trees on Guam shows that this cyanobacterium can produce BMAA using a highly selective and sensitive combination of methods. Similarly, Banack et al. (2007) detected BMAA in the same cyanobacterial genus using five different analytical platforms.

The major concern raised by Chernoff et al. (2017) mostly focused on the degree of association of cyanobacteria with BMAA (point 2). Their assessment (Chernoff et al. 2017) focused on the role of analytical methods with a division in the methods that detected BMAA (derivatized) *versus* those that largely did not detect BMAA (underivatized)––although Faassen et al. (2012) using an underivatized method did detect BMAA in all samples analyzed (Faassen et al. 2012). The analytical method employed may also reveal other issues with respect to BMAA detection. As BMAA is generally present at low concentration in environmental samples and may be at or slightly below the LOD, discrepancies in results may also arise from differences in these limits and the linear analysis range (Bishop and Murch 2020). BMAA was detected in cyanobacterial matrices in 95% of RPLC studies, but in only 25% of HILIC studies (Bishop and Murch 2020). This discrepancy likely arose from matrix effects or, unsuitable or improperly validated methods. Using a HILIC method, very low BMAA recoveries in a *Leptolyngbya* cyanobacterial strain (7–21%) were reported, while recoveries much greater than 100% were reported in zooplankton (*Daphnia magna*; 141%) (Faassen et al. 2016). Using the same method, Tymm et al. (2019) found recoveries < 10% in the cyanobacterium *Spirulina* (Tymm et al. 2019). However, with a validated RPLC method with AQC derivatization adequate recovery (between 104.8 and 107.5%) of BMAA was observed in a cyanobacterial matrix (*Spirulina*) and a recovery of between 94 and 106% was obtained in a cyanobacterial mat containing *Phormidium*, *Dapis*, and diatoms (Glover et al. 2015; Banack 2020). The higher sensitivity and higher molecular weight of derivatized BMAA may enable detection of low concentrations of BMAA in a matrix where salts, metals, carbohydrates, and other amino acids are abundant, whereas HILIC methods may not always be sensitive or reliable enough for such conditions (Baker et al. 2018; Tymm et al. 2019). Although unvalidated or insufficiently validated HILIC methods could have led to this discrepancy in results, some HILIC and RPLC methods that were validated also produced negative results.

Even though research into the factors which influence the production of BMAA by cyanobacteria is nascent, nitrogen appears to be a key driver in its production, with nitrogen reduction or removal resulting in an increase in its concentration in non-nitrogen-fixing cyanobacteria (Downing et al. 2011; Scott et al. 2014). Nitrogen starved *Microcystis* and *Synechocystis* cultures fed ^15^N-BMAA produced increased amounts of free cellular BMAA compared to nitrogen fed cultures, with ammonium resulting in an even more rapid depletion of BMAA than nitrate addition (Downing et al. 2011).

Another practical aspect of cyanobacterial BMAA investigations that requires clarification are differences between the assessment of cultured versus environmental samples of cyanobacteria (Cervantes Cianca et al. 2012; Fan et al. 2015; Monteiro et al. 2017). Although in environmental samples, variable biotic and abiotic conditions can exist, under culture conditions the choice of culture media may have a great effect on BMAA production, such as the very high nitrate concentration in BG11 medium (Bishop 2020). Therefore, modifications to culture medium or the use of multiple media types may be needed to determine under what conditions the production of BMAA may be possible in the strain under investigation.

The assessment of BMAA production from cyanobacteria, present within environmental samples (Metcalf et al. 2008, 2018), may also be influenced by the co-occurrence of other organisms that also have the capacity to produce BMAA (point 3) including diatoms, dinoflagellates, and chemoheterotrophic bacteria (Nunn and Codd 2019). Even the intestinal flora of humans has been hypothesized to be a potential source of BMAA (Nunes-Costa et al. 2020) and related hypotheses have emerged which implicate the human microbiota in neurodegenerative disease (Obrenovich et al. 2020). In recent studies, BMAA has been detected within several of these organisms including microbial biofilms, marine and freshwater diatoms, dinoflagellates, and zooplankton (Réveillon et al. 2016; Chatziefthimiou et al. 2018; Jungblut et al. 2018; Violi et al. 2019; Zguna et al. 2019; Metcalf et al. 2020; Bishop et al. 2020). This expanding field of research will provide interesting data to help us understand the complex interactions of non-protein amino acids in a variety of organisms and ecosystems. The finding that BMAA production may not be limited to cyanobacteria but be produced more broadly in other biological groups, would suggest that this neurotoxic amino acid could be detected more frequently within environmental samples.

## Misincorporation of BMAA into Proteins

Misincorporation of BMAA is only one of many mechanisms acknowledged as possibly contributing to triggering sporadic neurodegenerative diseases (Chiu et al. 2011; Arif et al. 2014) and for examples since 2017 see (Beri et al. 2017; Metcalf et al. 2017; D‘Mello et al. 2017; Potjewyd et al. 2017; Powers et al. 2017; Engskog et al. 2017; Michaelson et al. 2017; Díaz-Parra et al. 2017; Tan et al. 2018a, b; van Onselen and Downing 2018, 2019; Albano and Lobner 2018; Main and Rodgers 2018; Laugeray et al. 2018; Lepoutre et al. 2018; Pierozan et al. 2018, 2020; Gerić et al. 2019; Cheng et al. 2019; Pierozan and Karlsson 2019; Tedeschi et al. 2019; Diaz-parga et al. 2020; Li et al. 2020; Ndaru et al. 2020; Vallerga et al. 2020). Chernoff et al. (2017) presents multiple lines of evidence to refute that L-BMAA is a substrate for protein incorporation and we address these below.

It is now well established that amino acid analogues that are structurally similar to their cognate counterparts can be aminoacylated by relevant transfer RNA (tRNA) synthetases and subsequently incorporated into nascent polypeptides during protein synthesis (Rosenthal 1977; Noren et al. 1989; Rodgers et al. 2002; Wang and Schultz 2005; Hartman et al. 2006, 2007; Gurer-Orhan et al. 2006; Nangle et al. 2006; Rubenstein 2008; Dunlop et al. 2011; Chan et al. 2012; Oh et al. 2014; Shiozawa-West et al. 2015). tRNA synthetases discriminate based on size and charge; thus, amino acids that are able to fit into the active site pocket can be misacylated and mischarged (Lee et al. 2006).

Since structural similarities of some cognate amino acids means they are highly susceptible to misacylation, human tRNA synthetases have evolved editing mechanisms to hydrolyze incorrectly associated cognate amino acids (Klipcan et al. 2009; Moor et al. 2011).

Post-translationally modified forms of cognate amino acids, including oxidized amino acids, are also subject to misincorporation. For example, 3,4-dihydroxy-L-phenylalanine (DOPA) is an oxidization product of phenylalanine and tyrosine and is aminoacylated by the phenylalanyl- (Moor et al. 2011) and tyrosyl-tRNA synthetases (Ozawa et al. 2005) and inserted into proteins (Ozawa et al. 2005; Rodgers et al. 2006; Chan et al. 2012). *m*-tyrosine, (*meta*-tyrosine or 3-OH-phenylalanine), and *o*-tyrosine (*ortho*-tyrosine or 2-OH-phenylalanine) are oxidation products of phenylalanine and are aminoacylated by the phenylalanyl-tRNA synthetase (Gurer-Orhan et al. 2006; Moor et al. 2011) and inserted into proteins during synthesis.

A new level of complexity is introduced with non-protein amino acids for which ingestion is the most common route for exposure (Dunlop et al. 2014). For example, L-canavanine is contained in alfalfa, is a natural homologue of L-arginine, and is mis-acylated by the arginyl-tRNA synthetase, inserted into proteins, and creates aberrant canavanyl proteins (Akaogi et al. 2006). Azetidine-2-carboxylic acid (AZE) is contained in sugar beets (Rubenstein et al. 2009), *Convallaria* species (Fowden 1959), and garden beets (Rubenstein et al. 2006) and is structurally similar to proline and alanine (Song et al. 2017). AZE can misincorporate in place of proline (Fowden and Richmond 1963) in myelin basic protein (MBP) (Bessonov et al. 2010) and this has been hypothesised to be implicated in the pathogenesis of multiple sclerosis (MS) (Rubenstein 2008). Indeed, AZE is a dual-mimic that can be acylated by both the alanyl- and prolyl-tRNA synthetases and inserted into nascent polypeptides during protein synthesis (Song et al. 2017).

The hypothesis that L-BMAA can be misincorporated was based on the observation that free BMAA concentrations increase 10–240-fold following hydrolysis of cycad flour (Murch et al. 2004b), suggesting that environmental bioaccumulation might be a function of protein synthesis. Several publications have contended this hypothesis however, suggesting that accummulation of L-BMAA could equally be a function of non-covalent interactions, not misincorporation leading to covalent bonds, the latter of which refers to ribosomal-dependent incorporation. We propose it is likely there is a role for both mechanisms in BMAA accumulation in proteins, and protocols have been developed to distinguish between the two. First, to separate the free and protein-bound BMAA fractions, trichloroacetic acid (TCA) is added to the samples to precipitate the proteins. The resulting pellets are then thoroughly washed with multiple rounds of TCA followed by combinations of SDS and/or DTT to remove any non-covalently associated BMAA. Some groups (Okle et al. 2013; van Onselen et al. 2017) then purify the proteins using SDS-PAGE, then extract the bands and hydrolyze the pellets for BMAA quantitation. The process of acid hydrolysis cleaves peptide bonds and releases the covalently bound BMAA.

One experimental method used by Dunlop et al. (2013) and others (Gurer-Orhan et al. 2006) adopted to distinguish between misincorporation/covalent and non-covalent interactions of BMAA with proteins is the use of the protein synthesis inhibitor, cycloheximide (CHX). CHX targets the elongation phase of translation, so this intervention specifically targets ribosomal-dependent incorporation of L-BMAA.

The Chernoff et al. (2017) review is critical of data that shows that CHX reduces the incorporation of radiolabeled ^3^H-L-BMAA into proteins (Fig 2 in Dunlop et al. 2013); we presume because the authors infer that a global reduction in protein synthesis mediated by the presence of CHX will simultaneously reduce the amount of ^3^H-L-BMAA being incorporated into nascent polypeptides. To control for this, Dunlop et al. (2013) conducted concurrent incubations of MRC5 human fibroblasts with ^3^H-L-leucine (Fig. 2, open bars in Dunlop et al. 2013), a cognate amino acid known to be incorporated via protein synthesis. The addition of CHX to MRC5s incubated with ^3^H-L-leucine reduced the amount of radiolabel to virtually the same extent as ^3^H-L-BMAA, suggesting that the mechanisms for incorporation of these two amino acids were the same. The use of CHX also provides evidence for a role for new protein synthesis in the incorporation of L-BMAA into proteins (Gurer-Orhan et al. 2006). These observations support ribosomal-dependent incorporation as one mechanism involved in the association of BMAA with proteins.

Since the Dunlop et al. (2013) paper appeared in 2013, multiple studies in various models have been published reporting both covalent and non-covalent interactions of BMAA with proteins (see for example, Glover et al. 2014; van Onselen and Downing 2018). Dunlop et al. (2013) differentiated between the two mechanisms as described above. Briefly, Dunlop et al. (2013) incubated SH-SY5Y human neuroblastoma cells with ^3^H-L-BMAA in Hanks buffered salt solution (HBSS), precipitated proteins using TCA 10% and treated/washed them with SDS (2%), DTT (1 mM) or both SDS and DTT (Fig. 3 in Dunlop et al. 2013). DTT reduces sulfide bonds in proteins, and SDS is an anionic detergent that denatures and linearises proteins by disturbing non-covalent forces such as hydrophobic bonds. Thus, any BMAA that was not covalently bound in proteins should be liberated using this technique. No radiolabel was recovered following these denaturing steps, suggesting that the interaction of ^3^H-L-BMAA with protein in this model was not hydrophobic, electrostatic, or some other non-covalent association. However, free BMAA was liberated from proteins subjected to acid hydrolysis (6 M HCl, 110 °C, 16 h, Fig. 3 in Dunlop et al. 2013), a process which cleaves covalent bonds, providing further evidence for ^3^H-L-BMAA being covalently associated with proteins.

Further evidence of a role for protein synthesis in the interaction of ^3^H-L-BMAA with proteins in this model was provided by the use of the D enantiomer of serine as a negative control for incorporation (Fig. 4b in Dunlop et al. 2013). Human amino acyl tRNA-synthetases catalyze the attachment of L-amino acids to their cognate tRNA using a two-step mechanism in which the amino acid is first activated by ATP, then transferred to the tRNA, to form the aminoacyl-tRNA product (First 2011). Incorporation studies where L- and D-serine were used to inhibit the incorporation of ^3^H-L-BMAA show that only L-serine reduced the amount of ^3^H-L-BMAA in the proteins, thereby suggesting a role for ribosomal-dependent protein synthesis in the incorporation of ^3^H-L-BMAA.

Additional evidence for ^3^H-L-BMAA being misincorporated is presented in data showing the liberation of free BMAA when precipitated proteins are treated with the serine protease, Pronase E, or gas-phase acid hydrolysis (Fig. 3 in Dunlop et al. 2013). Both these techniques cleave covalent peptide bonds; Pronase E, enzymatically, and HCl via gas-phase hydrolysis. Thus, liberation of L-BMAA from proteins digested using a protease or acid-phase hydrolysis indicates covalent bonds are involved in the interaction of L-BMAA with proteins.

Taken together, these data from Dunlop et al. (2013) provide good evidence that one mechanism of L-BMAA interaction with proteins occurs via ribosomal-dependent protein synthesis leading to a covalent bond.

Other studies have also reported a role for protein synthesis in the association of L-BMAA with proteins. An autoradiographic study by Karlsson et al. (2009a) demonstrated that ^3^H-BMAA, administered intravenously to mice and frogs had a similar distribution pattern as protein-forming amino acids. The radioactivity was retained in tissues with high cell turn-over and/or protein synthesis, even after extraction with TCA, indicating that BMAA can be incorporated in or associated with proteins during synthesis (Karlsson et al. 2009a). Follow-up-studies using autoradiographic imaging and ultra-high performance liquid chromatography-tandem mass spectrometry (UHPLC-MS/MS) revealed a similar behavior of BMAA in rats (Karlsson et al. 2014).

Furthermore, and in a study cited by Chernoff et al. (2017), Xie et al. (2013) reported BMAA was associated with proteins following the administration of [14-C]-L-BMAA via bolus IV administration to mice. The authors measured brain distributions of total radioactivity in TCA-protein precipitates and supernatant from 5 min to 168 hrs (7 days) by autoradiography and liquid scintillation counting. They reported that the radioactivity (dpm/mg tissue) measured at 1, 3, and 7 days did not decrease over time and therefore concluded that BMAA was being misincorporated into cerebral proteins generating a pool of long half-life proteins (Xie et al. 2013).

While Dunlop and colleagues did not find evidence for non-covalent interactions of L-BMAA with proteins generated in cell culture, Glover et al. (2014) reported that approximately 50% of BMAA was liberated from proteins following protein denaturation using 1.5% SDS and 2 mM DTT. This group synthesized proteins in a cell-free system using components purified from *E. coli *(PUREexpress protein synthesis kit, New England Biolabs) with dyhydrofolate reductase (DHFR) and DNA from postmortem brain tissues of three human patients (control, Alzheimer’s disease and ALS) as templates. In concert with Dunlop et al. (2013), they also identified a role for covalent binding of L-BMAA evidenced by recovery of 50% of total BMAA from TCA-precipitated proteins following acid hydrolysis (6 N HCl, 110 °C, 16 h).

Glover’s work suggested L-BMAA also competes for incorporation at phenylalanine, proline, alanine, and glutamate sites, since when these amino acids (AAs) were separately excluded from the reaction mix, between 10 and 20% of the added BMAA was still incorporated (Fig. 3 in Glover et al. 2014). They detected L-BMAA incorporation even when all AAs were present in the reaction mix, and this was at a similar rate of relative incorporation as when L-serine was omitted from the reaction mix (Fig. 3 in Glover et al. 2014). This corresponds with data from Dunlop et al. 2013 (albeit in a different model), where the tritiated label of ^3^H-L-BMAA was detected in ~ 20% of proteins even when supplemented into complete medium (Fig. 4 in Dunlop et al. 2013). Further, the omission of L-serine and supplementation with ^3^H-L-BMAA resulted in recovery of the radiolabel in ~ 80% of the TCA-precipitable fraction. Taken together, these data suggest that L-BMAA can substitute for multiple amino acids, thereby amplifying its potential for neurotoxicity.

Regarding the contradictory findings for non-covalent association of L-BMAA with proteins from these independent labs, one aspect could be the use of different models by these two groups—cell free *versus *cell culture. In any case, and taken together, it seems likely there are at least two mechanisms involved in the “protein association” of L-BMAA—ribosomal-dependent protein synthesis leading to a covalent bond, and non-covalent, electrostatic interactions that are subject to disruption by anionic detergents and the breaking of sulphide bonds. These mechanisms are not mutually exclusive.

Nevertheless, the Chernoff et al. (2017) review raises some important issues regarding covalent and non-covalent interactions of BMAA, some of which have been addressed since 2017 (Dunlop and Guillemin 2019) and some that remain unresolved, and are addressed below.

One such unresolved issue involves the loss of BMAA signal in proteins following purification by SDS-PAGE. Okle et al. (2013) used autoradiography to detect [1-^14^C]-L-BMAA in proteins synthesized using a cell-free in vitro protein expression assay system (Kit #88858, Pierce/Thermo Scientific, Rockford, IL). The radiolabel was no longer detected following purification of the proteins by SDS-PAGE (Okle et al. 2013). The 2013 report from Okle et al. used a cell-free expression system containing pCFE-GFP mRNA and either ^14^C-L-BMAA or ^14^C-L-alanine as a control, and showed the presence of radiolabel in TCA-precipitated proteins (as measured by liquid scintillation counting) but the L-BMAA-radiolabel disappeared following protein purification by denaturing SDS-PAGE (Supplementary data, Figure 5 in Okle et al. 2013). It was difficult to pinpoint the data to which Chernoff et al. (2017) referred, since they incorrectly state that Okle’s BMAA-incorporation model was SH-SY5Y cell cultures, when instead it was an in vitro protein expression assay (Okle et al. 2013). Okle et al. (2013) were circumspect in their conclusions about loss of the label in their model and suggested that limitations of their method sensitivity might explain their negative results for BMAA misincorporation in SDS-PAGE purified proteins.

van Onselen and colleagues have published several studies reporting that BMAA is not misincorporated, primarily based on data from proteins extracted from cell cultures incubated with BMAA then purified using SDS-PAGE and run on HPLC-MS/MS or LC–MS. In one study (van Onselen et al. 2017), van Onselen et al. treated HeLa, HepG2, and Caco-2 cells with L-BMAA, precipitated proteins in 20% TCA, washed them twice more in 20% TCA to ensure all free amino acids were removed, purified them via SDS-PAGE, extracted, dried, and hydrolyzed them using 6 N HCl for 16 h at 110 °C, then analyzed them using mass spectrometry. HPLC-MS/MS of the AQC-derivatized amino acid samples did not reveal BMAA in any of the protein extracts at any of the exposure concentrations. However, free, non-protein-associated BMAA was detected in the cell lysates of all cells confirming BMAA uptake by the cells (van Onselen et al. 2017, p. 10, data not shown).

In support of their conclusion that BMAA is not misincorporated, the authors reported comparative toxicity of BMAA compared to what they refer to as “known misincorporating amino acid analogues” [L-*m*-tyrosine and L-4-fluorophenylalanine (FPA)] and showed that, unlike the known misincorporating analogues, BMAA did not induce concentration-dependent apoptosis or necrosis or affect the reduction of MTT (van Onselen et al. 2017, p. 10). van Onselen concluded that “in conjunction with the differences in cell viability and apoptosis/necrosis observed between the known misincorporating analogues and BMAA (we) strongly suggest that BMAA does not misincorporate into proteins in these human cell lines.” (van Onselen et al. 2017, p. 10). We respectfully suggest that some characteristics of the experimental design in this study may have contributed to these observed negative results. The issue of residual serine in cell culture, either as carry-over from the maintenance media or in the treatment media itself, has been extensively addressed in a recent review (Dunlop and Guillemin 2019). In this review, Dunlop and Guillemin (2019) describe the requirement to minimize residual serine as a way to maximize the likelihood of observing BMAA misincorporation in vitro. This includes thorough washing of cells using a serine-free medium such as HBSS or PBS prior to adding the treatment media (in our experience, three washes at least are required). van Onselen did not describe undertaking a wash in their methods, nor did they provide a sufficient description of their culture medium to ascertain if it is serine-free. While they did not observe BMAA misincorporation in this model, we do not find their evidence as it is presented, sufficient to dismiss the BMAA misincorporation hypothesis entirely.

In a subsequent paper, van Onselen conducted similar experiments, but this time in PC12 cells and using the arginine analogue and non-protein amino acid, canavanine. Determination of misincorporation was made using EZ:faast™ amino acid analysis kit for LC–MS (van Onselen et al. 2018), and the authors reported that “…BMAA misincorporation into the primary structure could not be demonstrated..” (van Onselen et al. 2018, p. 21). Curiously, these authors did report finding BMAA in the protein fraction of PC12 cells incubated with BMAA, but stated that there was no evidence to suggest that this association is the result of misincorporation (van Onselen et al. 2018).

The Chernoff et al. review (2017, p. 26) states that definitive proof of misincorporation of L-BMAA, “needs to involve LC–MS/MS evidence that it is part of a protein amino acid backbone in peptides derived from partial enzymatic protein hydrolysis.” One such study was undertaken by Beri et al. in 2017. This group conducted LC–MS/MS analysis of SILAC-labeled cellular and secreted proteins extracted from NSC34 cells incubated with L-BMAA, as well as synthetic proteins generated from a cell-free expression system. Detection of L-BMAA misincorporation was measured using a dynamic modification of + 13.0316 Da on serine residues for identification. The authors reported no evidence for L-BMAA incorporation in any of the proteins they examined; however, the cell culture medium contained 0.21 g/L of L-serine, which would have very likely inhibited any incorporation of L-BMAA. Previous in vitro (cell-free) studies examining the incorporation of analogues of *N*-methyl amino acids showed only moderate yields of translated peptides in the presence of very small amounts of the cognate amino acid (Hartman et al. 2007). In the case of L-BMAA and L-serine, the cognate amino acid (L-serine) is a much more efficient substrate for the seryl-tRNA synthetase; thus, even trace amounts of serine will likely outcompete for charging of L-BMAA (Hartman et al. 2007). Indeed, recent work from Han et al. (2020) examining the interaction of BMAA with human alanyl-tRNA synthetase (AlaRS) showed that while BMAA had a similar K_m_ to the known non-cognate AlaRS amino acid, serine, the K_max_ for BMAA was 40-fold lower. This means the rate of formation of an aminoacyl-adenylate (the first step in the charging of an amino acid onto a tRNA synthetase) for BMAA and AlaRS is comparatively slow. Thus, the presence of 0.21 g/L L-serine in the culture medium in Beri et al. (2017) likely outcompeted any BMAA for incorporation.

Using the same cell-free kit as Glover et al. (2014) but with a messenger RNA (mRNA) template as opposed to DNA, Beri et al. (2017) generated synthetic proteins, where the template was DHFR and a mutant form of superoxide dismutase (SOD1) that has been implicated in ALS, hSOD1 G93A. Beri et al. (2017) did not detect the incorporation of L-BMAA, but again, L-serine was present in all incubations, where the lowest ratio was 1:1 (L-BMAA:L-serine), and the highest 100:1. We have previously reported that ratios as low as 1 serine: 50 BMAA can reduce apoptosis in MRC5 human fibroblasts (Fig. 6C in Dunlop et al. 2013), suggesting even trace amounts of L-serine could block L-BMAA-mediated cytotoxicity. The possibility of contamination of commercial BMAA products with L-serine as an explanation for negative results in misincorporation experiments has also been addressed by Han et al. (2020). To eliminate any artificial effects from possible cognate amino acid contamination, these authors purified their BMAA with chromatography (Han et al. 2020).

It is worth pausing at this point to address what some readers may interpret as two conflicting positions in this section: (1) we assert that even trace amounts of L-serine in cell culture medium can contribute to a lack of detection of BMAA misincorporation in vitro; (2) we also assert that trace amounts of BMAA in vivo can lead to misincorporation even in the presence of endogenous serine. We do not see these statements as contradictory since (1) it has been demonstrated that even a single substitution of a canonical amino acid in vivo can lead to neurodegeneration (Lee et al. 2006 and see below), and (2) although discrete incorporation events are likely rare in vivo, they may lead to aggregated and proteolytically resistant proteins which can then seed other proteins to misfold (Frost and Diamond 2010) thereby amplifying the effect of the initial insult. In addition, while covalent incorporation of BMAA as a discrete event may be infrequent, there is evidence (Karlsson et al. 2014, 2015b; Xie et al. 2013) for the accumulation of BMAA, particularly in tissue and cells where cellular and/or protein turnover is low, such as neurons. Thus, the cumulative BMAA concentration in vivo, as a function of multiple discrete incorporation events, is likely to contribute to interference in cellular homeostasis (Okle et al. 2013). This hypothesis may also go towards explaining the well documented lag time between BMAA exposure and the onset of neurological disease (Caller et al. 2018).

One of the crucial differences between BMAA exposure in vitro and in vivo is obviously time, where an in vitro system attempts to condense a lifetime of BMAA exposure to several days or weeks, whereas long-term, chronic in vivo exposure can occur over decades or more. The inherent characteristics of BMAA misincorporation make re-creating in vivo exposure in cell culture particularly challenging and highlights the requirement for precise experimental design.

On the issue of BMAA substituting for serine, Chernoff et al.’s (2017, p. 24) critique of Dunlop et al. (2013), states, “The conclusion that levels of BMAA-associated proteins are dependent on the concentration of serine cannot be evaluated since no data were provided for other comparably tested amino acids.” Dunlop et al. (2013) reported these data as follows: “To determine if a specific protein amino acid was being replaced by BMAA, we examined competition between all 20 protein amino acids and ^3^H-BMAA for incorporation into cell proteins. We found that incorporation of ^3^H-BMAA into cell proteins was inhibited in the presence of L-serine in a concentration-dependent manner." (p. 3, Fig. 4A in Dunlop et al. 2013). Thus, Dunlop et al. (2013) did test all other amino acids and reported they had no impact on L-BMAA incorporation in the model. The data to which Chernoff et al. (2017) refer is presented in Fig. 4A of Dunlop et al. (2013) and shows a dose-dependent inhibition of ^3^H-L-BMAA incorporation into proteins (as measured by TCA precipitation and liquid-scintillation counting) in culture when increasing concentrations of L-serine are added to the culture medium. Chernoff et al. (2017) also critique a lack of standard curve to enable the reporting of absolute quantities of BMAA cited, however Dunlop et al. (2013) did not report or intend to report absolute values of L-BMAA.

There are several limitations of Dunlop et al. (2013) that warrant genuine criticism and these have since been addressed in a recent review (Dunlop and Guillemin 2019). Firstly, in most of the studies in Dunlop et al. (2013) BMAA was measured indirectly by following the tritiated label on the BMAA moiety. However, Dunlop et al. did report a direct measure of BMAA incorporation where BMAA is identified from acid-hydrolysis using daughter ions identified by tandem mass spectrometry (Fig. 5 in Dunlop et al. 2013).

Another limitation of Dunlop et al. (2013) which was missed by Chernoff et al. (2017) but instead was proposed by Dunlop and Guillemin (2019) is direct visualization of the interaction between L-BMAA and the corresponding amino acyl tRNA-synthetases. This has since been done by Han et al. (2020). These researchers used recombinant tRNA synthetases and demonstrated that BMAA was not a substrate for human serine-tRNA synthetase as proposed by Dunlop et al. (2013) but instead was charged by human alanyl-tRNA synthetase (AlaRS). Importantly, and relevant for the fidelity of protein translation, they reported that BMAA escaped proofreading to form a stable BMAA-tRNA^Ala^, inhibited activation of the cognate amino acid, alanine, and thwarted the editing functions of alanyl-tRNA synthetase (Han et al. 2020). The observation by Han et al. (2020) that BMAA simultaneously acts as a competitive inhibitor of the activation of alanine onto alanyl-tRNA synthetase, as well as inhibiting the editing function of alanyl-tRNA synthetase, provides a potential mechanism for BMAA interfering with the fidelity of protein translation, which may well contribute to the well characterized downstream cytotoxic effects of BMAA (Chiu et al. 2011). Although Han did not to observe codon-specific misincorporation of BMAA, they suggested that this might be a result of limitations in the resolution of their reporter, combined with a low fraction (3.5%) of BMAA-tRNA^Ala^ formation (Han et al. 2020). This area of investigation is nascent and more research will clarify this aspect of protein misincorporation.

Such low levels of reported mischarging of tRNA synthetases by non-cognate and non-protein amino acids naturally leads to the question of clinical significance. It has previously been reported that even low levels of misincorporation can trigger neurodegeneration (Lee et al. 2006). Indeed, it has been suggested that since errors in incorporation are so rare, then L-BMAA incorporation is biologically irrelevant. However, a recent study from Proctor et al. (2019) using molecular simulation and predictive energetic computation, showed substitution of one serine residue for L-BMAA resulted in dissociation of the SOD1 dimer, loss of metal binding, misfolding, aggregation, and the formation of oligomers.

Proctor et al. (2019) noted that while BMAA misincorporation may be rare, it could reasonably serve as a seed or nucleating event for further SOD1 aggregation via template-directed misfolding referred to as the templating mechanism of prion propagation. Indeed, such a mechanism has been demonstrated for misfolded SOD1 and TDP43, which induced pathological conformation in their natively folded counterparts and could also be transferred from cell to cell, propagating damage [for a review, see (Silverman et al. 2016)]. Evidence for misfolded proteins perpetuating pathology in proteinopathies is not new and has been extensively documented (Wells et al. 2019). For example, it has been shown that conditioned media from primary mixed cell cultures transfected with mutant FUS, or TDP43 or wild-type TDP43 (WtTDP43) can induce SOD1 misfolding in spinal cord cultures containing neurons and astrocytes prepared from human WtSOD1 transfected mice (Pokrishevsky et al. 2016).

Other groups have also examined the potential cytotoxicity of replacing serine residues with BMAA. Korn et al. (2020) synthesized amyloid beta (A$\beta$) peptides containing three BMAA substitutions: two for serine (Ser8BMAA and Ser26BMAA) and one for phenylalanine (Phe19BMAA) and addressed the impact of these BMAA substitutions on A$\beta$_40_ cytotoxicity and biophysical effects in the context of Alzheimer’s disease (Korn et al. 2020). In mouse primary neuron cell culture, they reported these substitutions increased cell death (as measured using lactate dehydrogenase release), induced activated caspase-3 (an indicator of apoptosis), and reduced neurite length, an early indicator of neuronal damage, when compared to wild-type A$\beta$_40_. Measurement of Aβ fibrillation kinetics showed only the Ser26BMAA substitution had any effect, increasing the elongation of the lag and fibrillation time. The authors noted their findings were in direct contradiction to Rauk (2018) who used molecular dynamics to examine the structure of A$\beta$_1–42_ where BMAA was substituted for the same 2 serine residues. Since Rauk (2018) did not identify the formation of any stable complexes of A$\beta$_1–42,_ they concluded that BMAA was likely not a factor in the pathology of Alzheimer’s disease.

A rigorous examination of the consequences of misacylation of amino acids was undertaken by Lee et al. (2006) who reported a direct link for misacylation of serine to alanyl-tRNA synthetase, misincorporation, and subsequent neurodegeneration in a mouse model of *sti* mutant mice (Sticky mouse). This group reported that a mutation in the gene encoding alanyl-tRNA synthetase caused an editing defect which manifested as selective sensitivity to serine in these animals. This single editing error and subsequent increase in misacylated tRNA^Ala^ resulted in the induction of endoplasmic reticulum stress, as indicated by increased expression of Grp78 and CHOP, upregulation of chaperones (HSC70 and HSP72), increased immunoreactivity for ubiquitin, leading to extensive Purkinje cell loss and neurodegeneration in these animals (Lee et al. 2006). This study elegantly demonstrated that very low levels of misincorporation, even of non-cognate (as opposed to non-protein) amino acids, can lead to neurodegeneration in vivo.

## Alternate Hypotheses of Causal Factors Related to ALS/PDC

In this review, we have provided evidence for a role for BMAA in triggering neurodegenerative illnesses but in no way do we assert this is the only environmental toxin or mechanism that contributes to the onset of these diseases. For example, regarding ALS, over 50 genes have now been identified as having an association with this disease, and while only a few are 100% penetrative, others likely increase risk when other risk factors are present (Al-Chalabi et al. 2014). With no single cause known for sporadic ALS or Alzheimer’s disease, most scientists agree a gene/environment interaction is likely, and further research is needed to tease out these relationships. However, given the accumulating evidence for a role for BMAA, we feel it unwise to rule this toxin out as a contributing factor, particularly as cyanobacterial blooms are increasing in frequency, size and duration with increasing global temperatures (Ho et al. 2019).

## Conclusions

In the last decade, scientific publications on the non-protein amino acid BMAA have dramatically increased as evidence has accumulated that BMAA-contaminated traditional foodstuffs caused a serious neurological disease among the indigenous Chamorro people of Guam. Four major pieces of evidence link BMAA exposure to Guamanian ALS/PDC.

- 1. Chronic dietary exposure to BMAA produces Guamanian ALS/PDC neuropathology in laboratory animals. In replicated experiments, ApoE4 homozygous vervets with chronic (140 days) dietary exposure to BMAA developed neurofibrillary tangles and $\beta$-amyloid plaques in some regions of the brain, similar to those that occur in the brains of Guamanian ALS/PDC patients (Cox et al. 2016). These vervets also developed microglial activation along their spines with dense proteinopathies of TDP-43, FUS, and other proteins associated with ALS/PDC (Davis et al. 2020). Control animals did not develop these neuropathologies.
- 2. These animals were dosed for 140 days with BMAA at a dose which approximates the lifetime exposure of a Guamanian ALS/PDC patient (Banack and Cox 2018).
- 3. Indigenous Chamorro villagers who died of Guamanian ALS/PDC had BMAA in some but not all their brain regions, while healthy control North Americans who did not suffer from progressive neurodegenerative illnesses had little or no detectable BMAA in any regions of their brains (Cox et al. 2003; Murch et al. 2004a, b; Pablo et al. 2009).
- 4. Cyanobacteria harbored by the roots of cycads produce BMAA (Cox et al. 2003; Banack and Cox, 2003b), a finding which has been extended to diverse taxa of cyanobacteria throughout the world (Cox et al. 2005; Esterhuizen and Downing 2008).

The possibility that exposure to BMAA in cyanobacterial blooms or dietary sources may prove to be a risk factor for other neurological diseases outside of Guam is currently being investigated by teams of interdisciplinary investigators in Australia, Canada, China, France, Norway, South Africa, Spain, Sweden, and other countries throughout the world. If sufficient evidence shows that BMAA exposure is a risk factor for serious brain diseases in the USA, primary monitoring and regulatory action will fall to the US Environmental Protection Agency. Unfortunately, Chernoff et al. (2017) concluded in their review that, “the hypothesis of a causal BMAA neurodegenerative disease relationship is not supported by existing data” (Chernoff et al. 2017, p. 1). If they are wrong in their assessment, the USA may fall behind other countries in taking simple steps to mitigate the results of BMAA exposure. The precautionary default principle, defined as “a cautious or pessimistic assumption that is used in the absence of adequate information and that should be replaced when such information is obtained” (Sandin et al. 2004), suggests that it would be prudent to protect citizens from BMAA exposures unless further research suggests otherwise.

## Abbreviations

AEG: *N*-(2-aminoethyl)glycine
ALS: Amyotrophic lateral sclerosis
ALS/PDC: Amyotrophic lateral sclerosis/parkinsonism dementia complex
sALS: Sporadic ALS
AOAC™: Association of Official Analytical Collaboration International
AQC: 6-Aminoquinolyl-N-hydroxysuccinimidyl carbamate
AZE: Azetidine-2-carboxylic acid
BAMA: β-Amino-N-Methyl-alanine
L-BMAA: β-*N*-Methylamino-L-alanine
BOAA: Beta-N-oxalylamino-L-alanine
CHOP: C/EBP homologous protein
CHX: Cycloheximide
DAB: 2,4-diaminobutyric acid
DHFR: Dyhydrofolate reductase
dpm: Disintegrations per minute
DTT: Dithiothreitol
ELISA: Enzyme-linked immunosorbent assay
EPA: Environmental Protection Agency
L-DOPA: 3,4-Dihydroxy-L-phenylalanine
FDA: US Food and Drug Administration
FTLD-U: Frontotemporal lobar degeneration with ubiquitin positive inclusions
GC-MS: Gas chromatography–mass spectrometry
GCxGC-TOFMS: Comprehensive two-dimensional gas chromatography time-of-flight mass spectrometry
Grp78: 78-KDa glucose-regulated protein
HBSS: Hanks buffered salt solution
HCl: Hydrochloric acid
HILIC: Hydrophilic interaction liquid chromatography
HPLC-FD: High performance liquid chromatography with fluorescence detection
HPLC-MS: High performance liquid chromatography with mass spectrometry
IHC: Immunohistochemistry
IUPAC: International Union of Pure and Applied Chemistry
FUS: Fused in sarcoma RNA-binding protein
LC: Liquid chromatography
LC-MS: Liquid chromatography–mass spectrometry
LC-MS/MS: Liquid Chromatography with tandem mass spectrometry
LOD: Limit of detection
LOQ: Limit of quantitation
MBP: Myelin basic protein
MND: Motor neuron disease
MS: Mass spectrometry
MS/MS: Tandem mass spectrometry
*m/z*: Mass to charge ratio
NFT: Neurofibrillary tangle
PND: Post-natal day
RPLC: Reversed phase liquid chromatography
SDS: Sodium dodecyl sulfate
SDS-PAGE: Sodium dodecyl sulfate polyacrylamide gel electrophoresis
SLE: Systemic lupus erythematosus
SPE: Solid phase extraction
TCA: Trichloroacetic acid
TDP43: TAR DNA-binding protein 43
tRNA: Transfer RNA
UHPLC: Ultra-high performance liquid chromatography
UPLC: Ultra performance liquid chromatography
UPLC-MS/MS: Ultra performance liquid chromatography with tandem mass spectrometry
US/NF: US Pharmacopoeia/National Formulary
